# Diagnostic and prognostic value of *SHOX2* and *SEPT9* DNA methylation and cytology in benign, paramalignant, and malignant ascites

**DOI:** 10.1186/s13148-016-0192-7

**Published:** 2016-03-01

**Authors:** Maria Jung, Svenja Pützer, Heidrun Gevensleben, Sebastian Meller, Glen Kristiansen, Dimo Dietrich

**Affiliations:** Institute of Pathology, University Hospital Bonn, Sigmund-Freud-Str. 25, 53127 Bonn, Germany; Department of Otolaryngology, Head and Neck Surgery, University Hospital Bonn, Bonn, Germany

**Keywords:** DNA methylation, Biomarkers, Ascites, Liquid biopsy, Cancer diagnosis, Cytology, Cell-free DNA, *SHOX2*, *SEPT9*

## Abstract

**Background:**

Cytology remains the gold standard for the detection of malignant cells in ascites. However, its sensitivity is limited. The aim of this study was to evaluate DNA methylation biomarkers for the differential diagnosis of benign (ascites in patients without malignancy), malignant (ascites in cancer patients directly caused by malignancy), and paramalignant (ascites in cancer patients caused by comorbidities but not by malignancy) ascites.

**Methods:**

A cohort of 283 patients (134 cancer patients, 149 patients with benign diseases) presenting with ascites was prospectively enrolled. Ascites was evaluated by means of cytopathological investigation and DNA methylation of *SHOX2* and *SEPT9* in the cell-free and cellular fraction. DNA methylation in bisulfite-converted DNA was determined using quantitative methylation specific real-time PCR. Cytopathological and DNA methylation results were evaluated with regard to diagnosis and overall survival (OS).

**Results:**

Patients with positive DNA methylation had a poor overall survival compared to methylation-negative patients (hazard ratio: HR = 1.97, *p* = 0.001). In multivariate survival analysis, DNA methylation was an independent prognostic parameter (*p* = 0.003) together with age (HR = 1.03, *p* < 0.001) and the presence of malignant disease (HR = 1.87, *p* < 0.001).

The combination of methylation with cytopathological analyses led to a 42 % increase in the detection rate of malignant ascites, resulting in 37 % positively diagnosed cancer patients and a specificity of 97 %. Among cancer patients, patients with DNA methylation-positive ascites showed an adverse clinical course (HR = 1.63, *p* = 0.039).

**Conclusions:**

DNA methylation testing adds diagnostic and prognostic information and might constitute an effective ancillary method for the differential diagnosis of malignant, paramalignant, and benign ascites.

**Electronic supplementary material:**

The online version of this article (doi:10.1186/s13148-016-0192-7) contains supplementary material, which is available to authorized users.

## Background

Ascites is defined as the pathological accumulation of fluid in the peritoneal cavity. It is the most frequent complication in patients with compensated cirrhosis with about 50 % of the patients developing ascites in a 10-year follow-up [1]. In addition to cirrhosis, ascites can be caused by malignant neoplasia, heart failure, tuberculosis, and pancreatitis [2]. Depending on the volume of the ascites, abdominal girth and body weight increases. Additionally, patients may suffer from dyspnea, abdominal pain, and anorexia [3]. Runyon et al. reported that malignancies account for 10 % of ascites [4]. The pathophysiologic mechanism of the development of malignant ascites is complex. An impaired lymphatic drainage combined with increased vascular permeability leads to the accumulation of protein and fluid in the peritoneal space [5]. Especially with increasing tumor burden, the lymphatic system fails to cope with the fluid accumulation [3].

Patients suffering from malignant ascites have a poor prognosis with the median overall survival being only 5.7 months from diagnosis [6]. Moreover, cancer patients may develop benign ascites due to comorbidities. This fluid accumulation does not contain tumor cells and is therefore termed “paramalignant.” Accordingly, the following three forms may occur:

- Benign ascites: Develops in patients without cancer due to non-cancerous conditions, i.e., liver cirrhosis. This ascites does not contain tumor cells.
- Paramalignant ascites: Develops in cancer patients due to comorbidities. This ascites does not contain tumor cells.
- Malignant ascites: Develops in cancer patients due to the invasion of the tumor into the peritoneal cavity. This ascites contains tumor cells.

In epithelial ovarian carcinoma patients, it was shown that patients with negative peritoneal cytology (paramalignant ascites) have a significantly better prognosis compared to patients with positive peritoneal cytology (malignant ascites) 10609494 [7]. The patients’ survival time strongly depends on the primary cancer site. However, 8 to 23 % of patients suffer from a carcinoma of unknown primary (CUP) [6, 8]. Among women, ovarian cancer is the most common malignancy causing ascites [9]. Ovarian cancer patients have a significantly better prognosis compared to patients with ascites associated with other primary malignancies [6].

An accurate and early detection of tumor cells in the ascites fluid is of strong clinical importance in different clinical settings. The discrimination between malignant and paramalignant ascites is of importance for clinical staging and influences treatment decisions. Ovarian cancer staged T1 for instance is classified as T1c in the presence of malignant ascites [10], and adjuvant chemotherapy is often recommended after surgery [11].

In clinical routine, investigation of the cause of ascites begins with obtaining the patients’ clinical history followed by a physical examination. Additional analyses include radiographic techniques or blood tests [12]. To distinguish malignant, paramalignant, and benign ascites, invasive techniques must be performed, and the obtained ascitic fluid is analyzed cytologically [3].

In patients with peritoneal carcinomatosis, the sensitivity of cytology amounts to approximately 97 %, making cytological analysis the gold standard for the diagnosis of malignancy in ascites samples [3]. However, regarding the results of cytological analyses irrespective of the existence of peritoneal carcinomatosis, sensitivity decreases to roughly 60 % [4, 13]. The sensitivity of cytological analysis is impeded by low tumor cell abundance in the ascitic sample and by the difficult differentiation between tumor and reactive mesothelial cells [14]. Biomarkers distinguishing between benign, paramalignant, and malignant ascites could potentially increase the sensitivity of ascitic fluid examination and might thereby eliminate the need for additional invasive techniques. Single tumor markers, i.e., CEA, CA 125, and CA 19-9 are not useful for diagnosing malignant ascites as their clinical performance has been described as insufficient [15, 16]. However, the application of a panel of tumor markers might yet improve diagnostic prospects [17].

DNA methylation markers have great potential for diagnosing cancer for several reasons: aberrant DNA methylation is a frequently observed characteristic of cancer cells [18–21], DNA itself has high chemical robustness, and DNA methylation marks are stably retained during mitosis and meiosis. Furthermore, several analytical techniques, e.g., methylation specific qPCR, allow for an accurate quantification of the respective biomarker [22–24]. Nevertheless, until now, only few studies have focused on the differentiation of malignant, paramalignant, and benign ascites based on DNA methylation biomarkers. Müller et al. showed significant prognostic impact of a panel of 15 DNA methylation markers in ascites and peritoneal washing samples of ovarian cancer patients [25]. Furthermore, Caceres et al. detected hypermethylation of *BRCA1* and *RASSF1A* in ascites samples and peritoneal washings from ovarian cancer patients [26].

Hypermethylation of the short stature homeobox 2 (*SHOX2*) or septin 9 (*SEPT9*) gene loci has been reported for several malignancies. *SHOX2* DNA methylation is a validated biomarker in bronchial fluid aspirates and allows for detection of lung cancer, even in patients for which cytopathological examination and bronchoscopy failed to detect malignancy [23, 27]. In addition, DNA methylation of *SHOX2* is a sensitive and specific biomarker in plasma in lung [28] and head and neck squamous cell carcinoma patients. *SEPT9* methylation has been detected at the onset of colorectal carcinogenesis [29] and is a validated plasma biomarker for colorectal cancer screening [30–32]. *SHOX2* and *SEPT9* DNA methylation are highly specific biomarkers for malignant pleural effusions and are a promising ancillary method in addition to cytological analysis potentially improving sensitivity and prognostic accuracy [22].

The aim of this study was to evaluate if *SHOX2* and *SEPT9* can increase the sensitivity of the detection of malignant cells in ascitic fluid. Furthermore, the prognostic value of both DNA methylation markers was investigated in order to deduce their potential for the clinical management of patients with ascites.

## Results

**Fig. 1:**
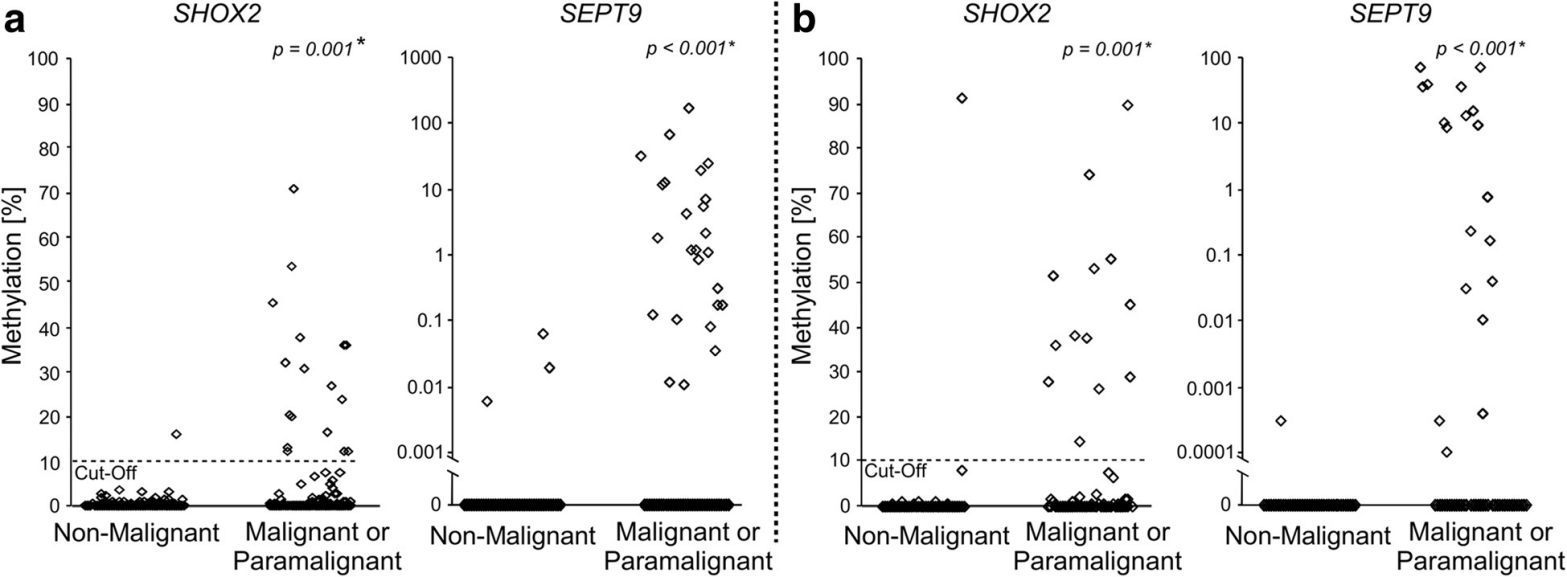
DNA methylation of *SHOX2* and *SEPT9* in ascitic samples from cancer and non-cancer patients. Comparison of *SHOX2* and *SEPT9* methylation of ascitic DNA from cancer patients and patients with exclusively non-malignant diseases determined by quantitative real-time PCR. Methylation cutoffs were introduced for *SHOX2* and *SEPT9* to dichotomize patient samples as *SHOX2* or *SEPT9* positive (above the cutoff) or negative (below the cutoff), respectively. The indicated *p* values refer to the Mann-Whitney *U* tests. **a** DNA methylation analysis of the cellular fractions of ascites samples (*n* = 283). **b** Methylation results of cell-free ascitic DNA (*n* = 162)

A total of 283 patients suffering from ascites were included in the study. A total of 134 patients had a known malignancy or were newly diagnosed with cancer during this study. An earlier study in which *SHOX2* and *SEPT9* methylation was determined in the cellular fraction of pleural effusions revealed an elevated *SHOX2* background methylation—even in patients without malignancies—while *SEPT9* methylation was solely found in cancer patients [22]. The background methylation of *SHOX2* necessitated the introduction of a methylation cutoff in order to classify samples as methylation positive (above cutoff) and methylation negative (below cutoff). In the present study, an elevated DNA methylation of *SHOX2* was also found in the cellular and cell-free fraction of benign ascites (Fig. 1). Hence, the cutoff previously established on pleural effusion (10 % *SHOX2* DNA methylation) was applied to the ascites samples analyzed in this study.

### *SHOX2* and *SEPT9* are prognostic biomarkers for overall survival in patients with ascites

**Fig. 2:**
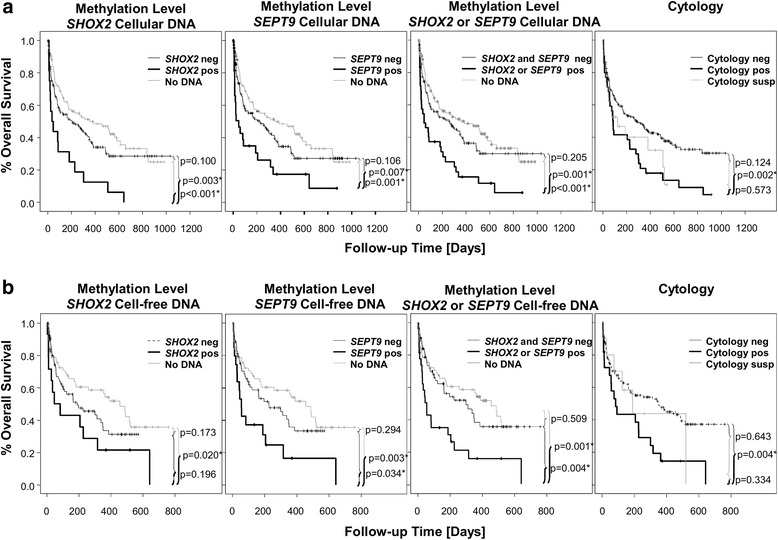
Kaplan-Meier survival analyses of cell-free and cellular DNA methylation analyses and cytology. Kaplan-Meier analysis of overall survival in 283 patients stratified by the cytological diagnosis or the cell-free and cellular DNA methylation status of *SHOX2* and *SEPT9.* The *p* values refer to the log-rank test. **a** Results of cellular DNA methylation analysis and cytology. **b** Results of cell-free DNA methylation analysis and cytology

Patients with malignant cells in ascites (malignant ascites) are expected to show an adverse clinical course compared to patients without malignant cells in ascites. The latter either presented with a non-malignant disease (benign ascites) or a malignant disease without tumor cells in the ascites (paramalignant ascites). Biomarkers allowing for the determination of the prognosis in patients with ascites might be powerful biomarkers for the discrimination between malignant ascites and ascites without tumor cells. Cytology is highly specific for the presence of tumor cells. Accordingly, in the present study, patients positive in cytological analyses had an adverse overall survival compared to patients with negative cytological results (Fig. 2a, *p* = 0.002). This confirmed the expectation that patients with malignant ascites have a worse clinical course than patients with benign and paramalignant ascites. However, the sensitivity of cytological analyses is limited. This, on the one hand, impairs the correct estimation of the specificity of the new biomarker test and on the other hand the number of paramalignant ascites. Due to the presence of occult and clinically non-significant tumors, i.e., prostate [33] and breast tumors [34], the number of occult tumors in the group of patients that are considered non-cancer patients is high and an accurate patient classification is hardly possible. Therefore, overall survival as clinical end point was used as a surrogate measure independent of the gold standard.

Indeed, an adverse clinical course was also found in patients with elevated DNA methylation of *SHOX2* and *SEPT9* in the cellular fraction of the ascites compared to methylation-negative patients (Fig. 2a, *p* = 0.003, *p* = 0.007, respectively). Hence, *SHOX2* and *SEPT9* methylation might be biomarkers for the presence of malignant cells in ascites. Moreover, patients with DNA amounts below the limit of quantification in the cellular fraction of ascites showed a significantly better prognosis compared to patients positive for *SHOX2* and *SEPT9* methylation (Fig. 2a, *p* < 0.001, *p* = 0.001, respectively). Kaplan-Meier analyses also revealed a trend towards a better prognosis in patients without quantifiable DNA amounts compared to methylation-negative patients, even though statistical significance was not reached (Fig. 2a, *SHOX2*: *p* = 0.100, *SEPT9*: *p* = 0.106). Positivity for either *SEPT9* or *SHOX2* methylation or both in the cellular ascites fraction was associated with a worse overall survival compared to methylation-negative patients or patients with low DNA amounts in the ascites sample (Fig. 2a, *p* = 0.001 and *p* < 0.001, respectively). Although not statistically significant, a trend for better overall survival in patients with low DNA amount in the cellular ascites fraction compared to patients negative for both *SHOX2* and *SEPT9* methylation was shown in Kaplan-Meier analyses (Fig. 2a, *p* = 0.205).

Furthermore, hypermethylation of the *SEPT9* locus in the cell-free fraction of ascites was a significant prognostic factor (Fig. 2b, *p* = 0.034) while *SHOX2* did not reach statistical significance (Fig. 2b, *p* = 0.196). Patients with low cell-free DNA (cfDNA) amounts showed a significantly better outcome compared to *SHOX2* or *SEPT9* methylation-positive patients (Fig. 2b, *p* = 0.020, *p* = 0.003, respectively). Patients positive for one or both methylation biomarkers had a significantly worse prognosis compared to methylation-negative patients or patients with low amounts of cfDNA (Fig. 2b, *p* = 0.004, *p* = 0.001, respectively). In general, the survival benefit of patients with low levels of cfDNA compared to patients with unmethylated *SHOX2*, *SEPT9*, or both was observed in Kaplan-Meier analyses but failed statistical significance (Fig. 2b, *p* = 0.173, *p* = 0.294, *p* = 0.509, respectively).

The results from Kaplan-Meier analyses were further confirmed in univariate Cox proportional hazards analyses (Tables 1 and 2). Patients positive for either *SEPT9* or *SHOX2* methylation or both in cellular (hazard ratio: HR = 1.97, *p* = 0.001) or cfDNA (HR = 2.17, *p* = 0.005) had a significantly worse overall survival rate compared to methylation-negative patients. Furthermore, patients with low DNA amounts in the cell-free and cellular fraction of ascites had a significantly better prognosis compared to methylation-positive patients (cellular: HR = 0.41, *p* < 0.001, cfDNA: HR = 0.39, *p* = 0.001) and a tendency towards better prognosis compared to methylation-negative patients (cellular: HR = 0.81, *p* = 0.212, cfDNA: HR = 0.85, *p* = 0.498). Moreover, cellular DNA methylation of *SHOX2* and/or *SEPT9* was shown to be an independent prognostic parameter (*p* = 0.003) together with age (HR = 1.03, *p* < 0.001) and the presence of a malignant disease (HR = 1.87, *p* < 0.001) in multivariate COX proportional hazards analysis (Table 1). The other clinicopathological factors (cytology and gender) were backward eliminated since they did not add additional significant prognostic information. In addition, methylation in the cell-free fraction of the ascites was an independent prognostic factor (*p* = 0.002) together with age (HR = 1.04, *p* < 0.001) while cytology, gender, and the presence of a malignant disease were eliminated due to the lack of additional significant information (Table 2).

**Table 1:** Univariate and multivariate Cox analyses on overall survival of ascites patients

	Univariate Cox analysis		Multivariate Cox analysis	
	Hazard ratio [95 % CI]	*p* value	Hazard ratio [95 % CI]	*p* value
Tumor (negative as reference)	2.24 [1.64–3.05]	<0.001	1.87 [1.35–2.59]	<0.001
Cytology (negative as reference)				
Positive	1.88 [1.27–2.78]	0.002		
Suspicious	1.54 [0.90–2.63]	0.117		
Gender (male as reference)	0.82 [0.60–1.12]	0.207		
Age (discrete variable)	1.04 [1.02–1.05]	<0.001	1.03 [1.02–1.05]	<0.001
Cellular *SHOX*2 + *SEPT9*		<0.001*		0.003*
Positive (negative as reference)	1.97 [1.30–2.97]	0.001	1.34 [0.87–2.07]	0.182
No DNA (negative as reference)	0.81 [0.58–1.13]	0.212	0.76 [0.54–1.07]	0.121
No DNA (positive as reference)	0.41 [0.26–0.64]	<0.001	0.57 [0.36–0.90]	0.015

Results of cellular DNA methylation analyses (*n* = 283). *p* values indicated by “*” refer to overall effect of the categorical variables irrespective of the reference levels

**Table 2:** Univariate and multivariate Cox analyses on overall survival of ascites patients

	Univariate Cox analysis		Multivariate Cox analysis	
	Hazard ratio [95 % CI]	*p* value	Hazard ratio [95 % CI]	*p* value
Tumor (negative as reference)	2.28 [1.48–3.53]	<0.001		
Cytology (negative as reference)				
Positive	2.10 [1.26–3.51]	0.005		
Suspicious	1.25 [0.54–2.88]	0.608		
Gender (male as reference)	0.76 [0.49–1.18]	0.217		
Age (discrete variable)	1.04 [1.02–1.06]	<0.001	1.04 [1.02–1.06]	<0.001
Cell-free *SHOX*2 + *SEPT9*		0.002*		0.002*
Positive (negative as reference)	2.17 [1.26–3.74]	0.005	2.22 [1.29–3.82]	0.004
No DNA (negative as reference)	0.85 [0.53–1.37]	0.498	0.89 [0.55–1.44]	0.628
No DNA (positive as reference)	0.39 [0.23–0.67]	0.001	0.40 [0.23–0.69]	0.001

Results of cfDNA methylation analyses (*n* = 162). *p* values indicated by “*” refer to overall effect of the categorical variables irrespective of the reference levels

### *SHOX2* and *SEPT9* are diagnostic biomarkers for malignancy in ascites

The diagnostic power of DNA methylation for the discrimination of cancer and non-cancer patients was investigated. *SHOX2* and *SEPT9* were hypermethylated in the cellular fraction (*n* = 283, *p* = 0.001, *p* < 0.052, respectively) and cfDNA (*n* = 162, *p* = 0.001, *p* < 0.001) in the ascitic fluid of cancer patients compared to patients with non-malignant diseases (Fig. 1). Both *SHOX2* and *SEPT9* were highly specific biomarkers showing 99 and 98 % specificity in the cellular fraction and 99 % specificity in the cell-free fraction of ascites, respectively. The cellular fraction of cancer patients was positive for *SHOX2* and *SEPT9* in 11 and 18 %, respectively (Table 3). CfDNA showed similar positivity rates for *SHOX2* and *SEPT9* (16 and 23 %). Positivity of cellular methylation analyses were significantly associated with cytological results (*SHOX2*: *p* = 0.002, *SEPT9*: *p* = 0.004, *SHOX2* and/or *SEPT9*: *p* = 0.001). Nevertheless, the combination of cytological analyses and both DNA methylation biomarkers in the cellular fraction increased the positivity rate from 26 to 37 % compared to cytological analyses alone. Similarly, the combination of cytology and DNA methylation analyses of cfDNA increased the positivity rate to 43 %. A significant association was only observed between *SEPT9* positivity and cytological analyses (*SHOX2*: *p* = 0.794, *SEPT9*: *p* = 0.033, *SHOX2* and/or *SEPT9*: *p* = 0.133). For available matched samples of cellular and cell-free ascitic DNA, the biomarker assays of both DNA samples and cytological analyses were combined leading to an increased positivity of 47 % at 95 % specificity.

**Table 3:** Clinical performance of the DNA methylation biomarkers *SHOX2* and *SEPT9* and cytology in ascites samples

Diagnostic method	Patients			Test result	
	All patients	Cancer patients	Non-cancer patients	Positivity	Specificity
Cytology	283	134	149	26 % (35/134)	100 % (149/149)
Cellular *SHOX2*	283	134	149	11 % (15/134)	99 % (148/149)
Cellular *SEPT9*	283	134	149	18 % (24/134)	98 % (146/149)
Cellular *SHOX2* + *SEPT9*	283	134	149	24 % (32/134)	97 % (144/149)
Cytology + cellular *SHOX2* + *SEPT9*	283	134	149	37 % (49/134)	97 % (144/149)
Cell-free *SHOX2*	162	81	81	16 % (13/81)	99 % (80/81)
Cell-free *SEPT9*	162	81	81	23 % (19/81)	99 % (80/81)
Cell-free *SHOX2* + *SEPT9*	162	81	81	31 % (25/81)	98 % (79/81)
Cytology + cell-free *SHOX2* + *SEPT9*	162	81	81	43 % (35/81)	98 % (79/81)
Cytology + cell-free *SHOX2* + *SEPT9* + cellular DNA *SHOX2* + *SEPT9*	162	81	81	47 % (38/81)	95 % (77/81)

Positivity rates and specificity of DNA methylation and cytological analyses and combinations, thereof

Methylation of *SHOX2* or *SEPT9* was detected in ascites samples of patients suffering from different malignancy entities including, among others, ovarian cancer, hepatic or pancreatic cancer, gallbladder or bile duct cancer, and non-Hodgkin lymphoma (Table 4, Additional file 1). The highest methylation level (165 %) of *SEPT9* was observed in a patient suffering from a cancer of unknown primary, apparently exceeding a methylation rate of 100 %.

**Table 4:** Clinical performance of DNA methylation and cytological analyses

	Diagnostic result (positive ascites from cancer patients)		
Primary tumor	Cellular or cell-free DNA methylation *SEPT9* or *SHOX2*	Cytology	Cellular or cell-free DNA methylation *SEPT9* or *SHOX2* or cytology
Digestive system	27/71 (38 %)	18/71 (25 %)	29/71 (41 %)
Stomach	2/6 (33 %)	2/6 (33 %)	3/6 (50 %)
Small intestine	0/2 (0 %)	0/2 (0 %)	0/2 (0 %)
Colon^a^	2/8 (25 %)	0/8 (0 %)	2/8 (25 %)
Rectum	0/2 (0 %)	0/2 (0 %)	0/2 (0 %)
Anus, anal canal, and anorectum^a^	1/1 (100 %)	0/1 (0 %)	1/1 (100 %)
Liver and pancreas^a^	11/31 (35 %)	7/31 (23 %)	12/31 (39 %)
Gallbladder and bile ducts^a^	11/21 (52 %)	9/21 (43 %)	14/21 (67 %)
Respiratory system	1/5 (20 %)	0/5 (0 %)	1/5 (20 %)
Head and neck squamous cell carcinoma ^a^	1/2 (50 %)	0/2 (0 %)	1/2 (50 %)
Lung and bronchus^a^	0/3 (0 %)	0/3 (0 %)	0/3 (0 %)
Pleural mesothelioma	1/1 (100 %)	1/1 (100 %)	1/1 (100 %)
Melanoma skin	0/1 (0 %)	0/1 (0 %)	0/1 (0 %)
Bones and joints	0/1 (0 %)	0/1 (0 %)	0/1 (0 %)
Breast^a^	1/6 (17 %)	1/6 (17 %)	1/6 (17 %)
Genital system	4/22 (18 %)	11/22 (50 %)	12/22 (55 %)
Uterine cervix and uterine corpus	0/2 (0 %)	0/2 (0 %)	0/2 (0 %)
Ovary^a^	5/18 (23 %)	11/18 (61 %)	12/18 (67 %)
Prostate^a^	0/2 (0 %)	0/2 (0 %)	0/2 (0 %)
Urinary System	2/6 (33 %)	2/6 (33 %)	3/6 (50 %)
Urinary bladder and renal pelvis^a^	1/4 (25 %)	1/4 (25 %)	1/4 (25 %)
Kidney	1/2 (50 %)	1/2 (50 %)	2/2 (100 %)
Brain and other nervous system	0/1 (0 %)	0/1 (0 %)	0/1 (0 %)
Lymphoma	4/17 (24 %)	0/17 (0 %)	4/17 (24 %)
Non-Hodgkin lymphoma^a^	4/11 (36 %)	0/11 (0 %)	4/11 (36 %)
Hodgkin lymphoma^a^	0/2 (0 %)	0/2 (0 %)	0/2 (0 %)
Myeloma^a^	0/4 (0 %)	0/4 (0 %)	0/4 (0 %)
Other and unspecified primary sites	1/3 (33 %)	2/3 (67 %)	2/3 (67 %)

Tumor (organ)-specific performance of the developed assay and cytology. In a retrospective cohort study including ascites from 283 patients with suspected malignant disease and 134 patients with histological confirmed primary cancer. For more detailed information on DNA methylation results view Additional file 1. Patients indicated by ^a^suffer from more than one primary tumor. For detailed information view Additional file 2

### *SHOX2* and *SEPT9* are prognostic biomarkers for overall survival in cancer patients with ascites

**Fig. 3:**
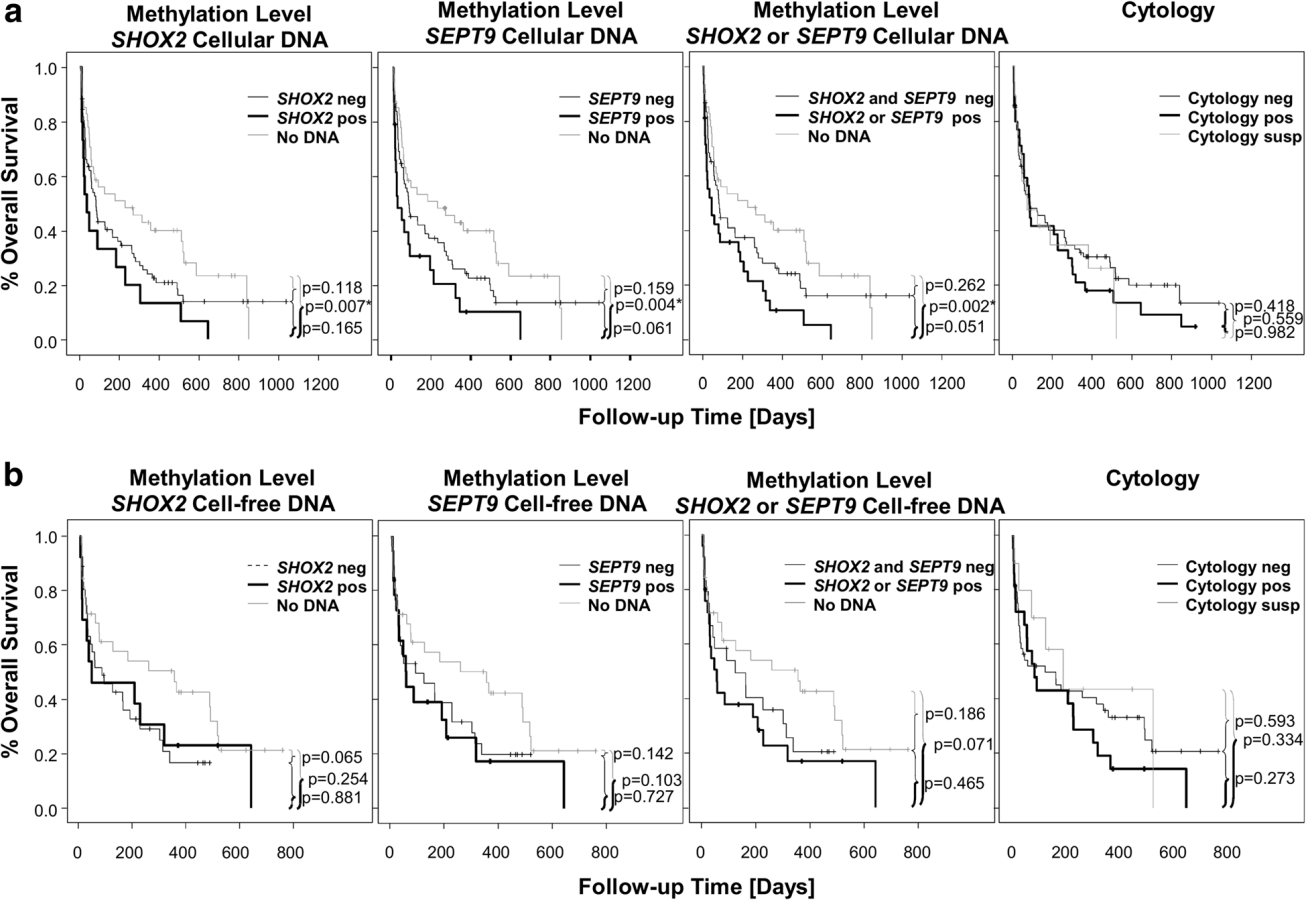
Kaplan-Meier survival analyses of cancer patients stratified by cell-free and cellular DNA methylation and cytology. The *p* values refer to the log-rank test. **a** Results of cellular DNA methylation analysis and cytology (*n* = 134). **b** Results of cell-free DNA methylation analysis and cytology (*n* = 81)

The diagnostic benefit in addition to the prognostic value of DNA methylation in cancer and non-cancer patients indicates that *SHOX2* and *SEPT9* methylation is a biomarker for an advanced malignancy. Patients with paramalignant ascites are likely to present with cancer at an earlier stage without involvement of the peritoneal cavity. Hence, patients’ survival is a potential surrogate measure for the discrimination between malignant and paramalignant ascites. Such a surrogate measure is useful since the gold standard (cytology) is limited regarding sensitivity, therefore leading to an apparent lower specificity of a new biomarker when comparing the new biomarker to the gold standard. Thus, the capability of *SHOX2* and *SEPT9* methylation to distinguish between malignant and paramalignant ascites was evaluated in a subgroup of cancer patients by comparing the survival in positive versus negative patients. Kaplan-Meier analysis of overall survival did not show significant prognostic impact of either methylated *SHOX2* (cellular DNA: *p* = 0.165; cfDNA: *p* = 0.881) or methylated *SEPT9* (cellular DNA: *p* = 0.061; cfDNA: *p* = 0.727) or cytology (*p* = 0.418) (Fig. 3). However, patients positive for either or both *SEPT9* and *SHOX2* methylation showed a trend towards worse prognosis compared to methylation-negative patients, particularly when analyzing the cellular fraction (*p* = 0.051) (Fig. 3a) although statistical significance was not reached. However, *SHOX2* and/or *SEPT9* methylation-positive patients showed a significantly lower overall survival in the analysis of the cellular DNA (*p* = 0.002, Fig. 3a) and a trend towards poor outcome when analyzing cfDNA compared to patients with low DNA amounts in the ascites samples (*p* = 0.071, Fig. 3b). Furthermore, patients with low cfDNA amounts tended to have a benefit with regard to overall survival compared to patients negative for *SHOX2* (cfDNA: *p* = 0.065, Fig. 3b).

In univariate COX proportional hazards analysis, cancer patients positive for *SHOX2* and/or *SEPT9* methylation showed a significantly worse outcome compared to patients with methylation-negative cellular DNA (HR = 1.63; *p* = 0.039, Table 5). The prognosis of patients with low DNA amounts in the cellular fraction of ascites was significantly better compared to patients positive for *SHOX2* and/or *SEPT9* methylation (HR = 0.47; *p* = 0.004 Table 5). Similarly, low amounts of cfDNA appeared to result in better prognosis compared to methylation-positive patients, even though statistical significance was missed (HR = 0.55; *p* = 0.060 Table 6). Multivariate COX proportional hazards analysis showed cellular (*p* = 0.017, Table 5) but not cfDNA methylation (Table 6) to be of independent prognostic impact together with age. Other clinicopathological factors like the cytological result and gender were eliminated due to their insignificant additional prognostic value.

**Table 5:** Univariate and multivariate Cox analyses on overall survival of cancer patients

	Univariate Cox analysis		Multivariate Cox analysis	
	Hazard ratio [95 % CI]	*p* value	Hazard ratio [95 % CI]	*p* value
Cytology (negative as reference)				
Positive	1.20 [0.78–1.86]	0.401		
Suspicious	1.20 [0.68–2.12]	0.528		
Gender (male as reference)	0.75 [0.51–1.11]	0.153		
Age (discrete variable)	1.03 [1.01–1.05]	0.002	1.03 [1.01–1.05]	0.002
Cellular *SHOX*2 + *SEPT9*		0.014*		0.017*
Positive (negative as reference)	1.63 [1.02–2.60]	0.039	1.36 [0.84–2.19]	0.211
No DNA (negative as reference)	0.76 [0.48–1.21]	0.250	0.64 [0.40–1.03]	0.065
No DNA (positive as reference)	0.47 [0.28–0.78]	0.004	0.47 [0.28–0.79]	0.005

Results of cellular DNA methylation analyses (*n* = 134). *p* values indicated by “*” refer to overall effect of the categorical variables irrespective of the reference levels

**Table 6:** Univariate and multivariate Cox analyses on overall survival of cancer patients

	Univariate Cox analysis		Multivariate Cox analysis	
	Hazard ratio [95 % CI]	*p* value	Hazard ratio [95 % CI]	*p* value
Cytology (negative as reference)				
Positive	1.37 [0.78–2.41]	0.275		
Suspicious	0.82 [0.34–1.96]	0.657		
Gender (male as reference)	0.69 [0.41–1.16]	0.217		
Age (discrete variable)	1.03 [1.00–1.06]	0.022	1.03 [1.00–1.06]	0.022
Cell-free *SHOX*2 + *SEPT9*		0.161*		
Positive (negative as reference)	1.21 [0.64–2.31]	0.557		
No DNA (negative as reference)	0.67 [0.35–1.28]	0.222		
No DNA (positive as reference)	0.55 [0.30–1.03]	0.060		

Results of cfDNA methylation analyses (*n* = 81). *p* values indicated by “*” refer to overall effect of the categorical variables irrespective of the reference levels

## Discussion

This study reveals that DNA methylation of *SHOX2* and *SEPT9* in ascites are significant prognostic biomarkers for overall survival independent of age, gender, cytological analysis, and the presence of a malignant disease. Patients positive for cellular or cfDNA methylation are shown to have a significantly worse overall survival compared to methylation-negative patients. As patients with malignant ascites are expected to have worse overall survival compared to patients with ascites due to benign conditions, it can be assumed that the methylation assay allows for detection of malignancy in ascites. The capability of the DNA methylation assay was evaluated with respect to the differential diagnosis of ascites. In this study, both biomarkers showed a specificity of 98 to 99 % while positivity of *SHOX2* or *SEPT9* DNA methylation in cancer patients was rather low ranging from 11 to 23 %. To avoid issues of multiple testing due to low patient numbers, we transferred the cutoff established on pleural effusions to the ascites samples (10 % *SHOX2* methylation [22]). To allow for a higher specificity of *SEPT9*, a cutoff of 0.1 or 0.01 % could be introduced for cellular or cfDNA. However, this would decrease the sensitivity of *SEPT9* methylation. Vice versa, the cutoff for *SHOX2* methylation could be set to 5 % leading to lower specificity but higher sensitivity. Therefore, the adaption of cutoffs ultimately needs to be validated in a larger cohort. Furthermore, this study showed increased positivity rates for the combination of the cellular or cell-free methylation analyses with cytology compared to the respective single analyses. Thus, the analysis of *SHOX2* and *SEPT9* DNA methylation might represent a promising ancillary method in addition to cytological analyses. Furthermore, it appears beneficial to quantify the methylation not only in the cellular but also in the cell-free ascitic fraction. Tumors have been reported to release free-circulating DNA most likely due to cellular apoptosis or necrosis [35, 36]. The detection of tumor-specific cfDNA has previously been established for cancer diagnostic and prognostics [28, 32, 37]. Nevertheless, the assignment of the measured cell-free tumor DNA either to DNA shed into the peritoneal cavity by an intra-abdominal tumor, free peritoneal cancer cells, cancer cells of a peritoneal carcinomatosis, or free-circulating plasma DNA is unfeasible. Thus, the definition of an ascites sample positive for cfDNA methylation as malignant may be inappropriate.

In general, the discrimination of true- and false-negative results depends on the gold standard, namely cytology. As the sensitivity of cytological analysis is limited, the portion of paramalignant ascites representing true negative results remains unclear. Paramalignant ascites was defined herein as ascites samples of cancer patients which do not contain tumor cells. Due to the lack of alternative methods providing a higher sensitivity than the current gold standard, the calculation of a negative predictive value is not trivial. Exclusively, a method diagnosing malignant ascites with 100 % sensitivity and 100 % specificity would allow for the correct interpretation of the methylation and the cytological analysis. We defined a negative methylation result in an ascitic sample of a cancer patient as false negative irrespective of the cytological result or existing comorbidities. Due to this limitation, the reported positivity of both the cytological as well as the methylation analyses is most likely underestimated. The high frequency of simultaneous medical conditions in cancer patients indicates that a high number of ascites is paramalignant and likely caused by a comorbidity rather than by free cancer cells in the peritoneal cavity or a peritoneal carcinomatosis. In detail, for a patient suffering from cancer and cirrhosis, the question whether portal hypertension and thus increased fluid entry from blood vessels to the peritoneal cavity caused ascites, or whether intraperitoneal cancer cells lead to an increased vascular permeability and impaired lymphatic drainage, is not trivial.

Among cancer patients, patients suffering from malignant ascites are expected to have a worse prognosis compared to patients with paramalignant ascites. Cancer patients positive for *SHOX2* or *SEPT9* cellular DNA methylation have a significantly worse prognosis. However, in single Kaplan-Meier analysis, neither *SHOX2-* nor *SEPT9-*positive patients show a significantly worse outcome compared to methylation-negative patients. Same holds true for single or combined *SHOX2* and *SEPT9* methylation analysis of cfDNA. It can be speculated that an increased cohort size leads to significant survival differences in cell-free analysis or single Kaplan-Meier analysis of *SHOX2* or *SEPT9*. Furthermore, a high prognostic impact of the primary tumor has been observed in ascites studies [6, 38]. Due to strong differences in mortality, e.g., ovarian cancer compared to pancreatic cancer, the additive prognostic value of *SHOX2* and *SEPT9* might be diminished. Hence, the introduced DNA methylation biomarker might be prognostically promising within a population of patients suffering from the same primary cancer entity. This hypothesis therefore needs to be validated in a study with higher statistical power and larger population sizes of patients with the same primary tumor entity. However, the prognostic impact of cellular methylation indicates that the assay allows for the discrimination of malignant and paramalignant ascites. Thus, this assay represents a suitable adjunct to cytological analysis as the latter has no significant prognostic impact on cancer patient survival in the conducted study.

Tumor DNA was detected in ascites samples of patients suffering from cancer of the digestive system, ovarian cancer, or lymphoma, among others. The positivity of both DNA methylation markers in ascites caused by different cancer entities confirms the results of DNA methylation analyses of *SHOX2* and *SEPT9* in the cellular fraction of pleural effusions. Both biomarkers are potentially applicable in different cancerous settings.

In addition, this study revealed that patients with low amounts of DNA in the cellular or cell-free fraction of ascites have a benefit with regard to overall survival, especially compared to methylation-positive patients. This finding is in concordance with studies reporting that increased amounts of cfDNA analyzed in serum or plasma correlates with the presence of malignancies and is associated with adverse outcome [39–43]. However, it cannot be excluded that low DNA amounts in ascites are due to the processing workflow of samples. In the conducted study, ascites samples were analyzed for DNA methylation 2 weeks after completion of the pathological diagnosis. This storage step might lead to the degradation of cells and cell-free DNA. Samples with low DNA amounts were defined as negative for the estimation of positivity rates. As the gold standard method cytology is included in the test, samples with low DNA amount are interpreted as samples with available cytology report and without DNA methylation results. Nevertheless, this definition causes an underestimation of sensitivity, as ascites samples of cancer patients with low DNA amounts are considered false-negative specimens.

Although repeated analyses by different experienced cytopathologists are hardly manageable in clinical routine, the sensitivity of cytological analyses may be improved by a reference cytopathological analysis. Increasing the sensitivity of cytological analyses might accordingly diminish the additive value of DNA methylation analyses. The analysis of DNA methylation biomarkers in contrast is highly robust and reproducible [22, 23] and does not necessitate highly experienced analysts. Furthermore, the detection of tumor DNA based on methylation quantification may benefit from gene locus amplification. An amplification of the *SHOX2* or *SEPT9* locus increases sensitivity compared to cell-based methods, i.e., cytology as four or more methylated copies of the *SHOX2* or *SEPT9* locus per tumor cell could exist. Indeed, a correlation between *SHOX2* methylation and amplification was shown in lung cancer tumors [44]. The same scenario is conceivable for *SEPT9* located on 17q25. An isochromosome 17q has been reported to be a frequently present in leukemia and solid tumors [45]. This isochromosome is characterized by a duplication of the long arm (q) including the *SEPT9* locus. Locus amplification of *SEPT9* or *SHOX2* without amplification of the reference gene (*ACTB*) can lead to methylation levels apparently above 100 %.

In summary, the combination of cfDNA analyses with cellular DNA methylation analyses or cytology resulted in an improvement of prognostic and diagnostic information. This may indicate that the additive value of cell-free analyses arises from detection of free DNA circulating in plasma and is not restricted to DNA of cells residing in the peritoneum.

## Conclusions

The DNA methylation biomarkers *SHOX2* and *SEPT9* are of diagnostic and prognostic value in ascites. The methylation of *SHOX2* and *SEPT9* of cellular and cell-free DNA was shown to be of additive diagnostic value to cytological analyses. Furthermore, it is beneficial to quantify the methylation not only in the cellular but also in the cell-free ascitic fraction. A significantly shortened overall survival was shown for patients positive for cellular *SHOX2* or *SEPT9* methylation. The methylation of cell-free or cellular DNA was shown to have a prognostic impact independent of age, gender, cytological analysis, and the presence of a malignant disease. Thus, DNA methylation of *SHOX2* and *SEPT9* should be analyzed as an adjunct to cytological analyses in the future as it improves the diagnosis of malignant ascites and is also promising in a prognostic setting.

## Methods

### Ethics, consent, and permissions

The study has been approved by the Institutional Review Board (IRB) at the University Hospital of Bonn (vote no. 141/13).

### Patients

Ascites samples from patients under investigation for suspected cancer at the University Hospital Bonn between 11/2012 and 02/2015 were included in this study. DNA methylation of *SHOX2* and *SEPT9* was measured in the cellular fraction of ascites fluid samples of 283 patients (134 cancer patients, 149 patients with exclusively non-malignant diseases) in a cohort study. Methylation of cell-free DNA was analyzed in 162 matched patient samples (81 cancer patients, 81 non-cancer patients). Patients’ characteristics are summarized in Table 7. Patients were considered to have developed ascites due to a non-cancerous condition if they did not have any evidence of cancer within the last 15 years. Detection of malignancy was performed by histological analysis based on biopsy or surgical specimens. Cytospins or smear preparations from ascitic fluid were stained by HE, PAS, PAP, and MGG staining for cytopathological analysis. Cell blocks were prepared in case of high cell numbers, and immunohistochemical staining of, e.g., thrombomodulin, TTF-1, and BerEP4 was performed. After completion of the routine diagnostics, ascitic fluid specimens were fixed with equal volume of Saccomanno’s fixative and centrifuged at 4.000×*g* at 23 °C. The pellets were dissolved in 1 ml of Saccomanno’s fixative and stored at room temperature. The supernatant was stored at −20 °C.

**Table 7:** Characteristics of the patient population

	Total	Cancer patients	Non-cancer patients
Age	283 (100 %)	134 (100 %)	149 (100 %)
≤50 years	57 (20 %)	20 (15 %)	37 (25 %)
51–60 years	67 (24 %)	26 (19 %)	41 (28 %)
>60 years	159 (56 %)	88 (66 %)	71 (48 %)
Median age (years)	62	67	60
Age range (years)	23–87	39–87	23–87
Follow-up			
Death	99 (35 %)	61 (46 %)	38 (26 %)
Alive	184 (65 %)	73 (54 %)	111 (74 %)
Mean follow-up (days)	162	141	180
Median follow-up (days)	59	56	83
Range (days)	0–832	0–832	0–774
Gender			
Female	121 (43 %)	62 (46 %)	59 (40 %)
Male	162 (57 %)	72 (54 %)	90 (60 %)
Non-malignant disease			
Hepatic failure	180 (63 %)	49 (36 %)	131 (88 %)
Gastrointestinal disease	104 (37 %)	36 (27 %)	68 (46 %)
Cardiac disease	80 (28 %)	33 (24 %)	47 (32 %)
Renal failure	47 (17 %)	22 (16.%)	25 (17 %)
Hepatitis A, B, C, D, or E, or autoimmune disease	46 (16 %)	18 (13 %)	28 (19 %)
Hepatorenal syndrome	40 (14 %)	11 (8 %)	29 (19 %)
Portal hypertension	25 (9 %)	4 (3 %)	21 (14 %)
Lung diseases	24 (8 %)	13 (9 %)	11 (7 %)
Sepsis	23 (8 %)	7 (5 %)	16 (11 %)
Hepatic encephalopathy	20 (7 %)	5 (4 %)	15 (10 %)
Pneumonia	16 (6 %)	5 (4 %)	11 (7 %)
Portal vein thrombosis	15 (5 %)	7 (5 %)	8 (5 %)
Pancreatitis	14 (5 %)	2 (1 %)	12 (8 %)
Peritonitis	14 (5 %)	2 (1 %)	12 (8 %)
Hemic disease	12 (4 %)	3 (2 %)	9 (6 %)
Others (benign tumors, urologic diseases, etc.)	10 (4 %)	4 (3 %)	6 (4 %)
Cytology result			
Positive	35 (12 %)	35 (26 %)	0 (0 %)
Negative	226 (80 %)	80 (60 %)	146 (98 %)
Suspicious	22 (8 %)	19 (14 %)	3 (2 %)

Clinical data of 283 patients (134 cancer patients, 149 non-cancer patients) included in the study

### Sample preparation

DNA extraction and DNA bisulfite conversions of the cellular fractions of the ascites fluid samples were performed using the innuCONVERT All-In-One Kit (Analytik Jena, Jena, Germany) as described earlier [22, 46]. In order to quantify methylation of cfDNA, extraction by polymer-based enrichment and bisulfite conversion of cell-free ascitic DNA was performed based on the innuCONVERT Bisulfite Body Fluids Kit (Analytik Jena, Jena, Germany) as previously reported [24]. If the supernatant (cell-free DNA) as well as the sediment (cellular DNA) of an identical ascites sample of a patient were available, both fractions were analyzed and defined as matching samples.

### Real-time PCR quantification of *SHOX2* and *SEPT9* DNA methylation

Quantification of *SHOX2* and *SEPT9* DNA methylation via real-time PCR was performed as previously described [22]. *SHOX2* and *SEPT9* were quantified in a methylation specific manner, whereas quantification of the β-actin gene (ACTB) served as a reference standard for total DNA input irrespective of the methylation status. Thresholds and baselines were defined as follows: 0.015 (threshold *SHOX2*), 0.01 (threshold *SEPT9*), 0.02 (threshold *ACTB*), and 3-24 (baseline). Each sample was analyzed in triplicate.

### Data evaluation and statistical analysis

Samples were included in the analysis when the median of the CT values met the following quality criterion: *CT*_Sample/ ACTB_ ≤ 31.5, or *CT*_Sample/ *SHOX*2_ ≤ 35, or *CT*_Sample/ *SEPT*9_ ≤ 40 [22]. Relative methylation values for each sample were determined using the ΔΔCT method adapted for DNA methylation analyses as previously described [22, 24, 47]. Samples were considered to have low DNA amounts including tumor DNA when CT values did not meet the predefined quality criterion, and methylation levels were defined as 0 %. In order to minimize false-positive results, a methylation cutoff was assigned for *SHOX2*. Thus, the quantitative results of *SHOX2* DNA methylation levels were transformed into qualitative results as samples showing a relative *SHOX2* methylation level above the cutoff were classified as positive and all others were classified as *SHOX2* negative, respectively.

Comparison of *SHOX2* and *SEPT9* methylation levels of cancer and non-cancer patients was performed using the Mann-Whitney *U* test. Linear-by-linear association of the chi-square statistic was performed to assess an association between methylation and cytological analyses. Univariate COX proportional hazards analyses and Kaplan-Meier analyses and log-rank tests were performed to assess a putative prognostic value of DNA methylation biomarkers and cytology. Multivariate COX proportional hazards analyses with backward elimination (Wald) were performed to assess a putative-independent prognostic value of DNA methylation analyses. *p* values <0.05 were considered as significant. All statistical analyses were performed using the SPSS software version 21 (IBM, Armonk, NY, USA).

### Availability of supporting data

The data sets supporting the results of this article are included within the article and its Additional files 1 and 2.

## Additional files

Additional file 1: Table S1. Clinical performance of DNA methylation analyses in detail. Tumor (organ) specific performance of the developed assay in a retrospective cohort study comprised of ascites from 283 patients with suspected malignant disease including 134 patients with histological confirmed primary cancer. Cell-free DNA was extracted from 162 patients including 81 patients with malignant diseases. (DOC 96 kb) Additional file 2: Table S2. Specification of 25 cancer patients suffering from more than one primary tumor. Other existing primary tumors are listed for patients suffering from more than one primary tumor. (DOC 76 kb)
