# Supplementary material for: Diagnostic and prognostic value of SHOX2 and SEPT9 DNA methylation and cytology in benign, paramalignant, and malignant ascites
Source: Clin Epigenetics. 2016 Mar 1;8:24. doi: 10.1186/s13148-016-0192-7 (PMC4774089; doi:10.1186/s13148-016-0192-7)
Supplement: Additional file 1: Table S1. — Clinical performance of DNA methylation analyses in detail. Tumor (organ) specific performance of the developed assay in a retrospective cohort study comprised of ascites from 283 patients with suspected malignant disease including 134 patients with histological confirmed primary cancer. Cell-free DNA was extracted from 162 patients including 81 patients with malignant diseases. (DOC 96 kb) [file 13148_2016_192_MOESM1_ESM.doc]

**Additional File 1**: **Clinical performance of DNA methylation analyses in detail.** Tumor (organ) specific performance of the developed assay in a retrospective cohort study comprised of ascites from 283 patients with suspected malignant disease including 134 patients with histological confirmed primary cancer. Cell-free DNA was extracted from 162 patients including 81 patients with malignant diseases.

|  | **Diagnostic result (positive ascites from cancer patients)** | | | |
| --- | --- | --- | --- | --- |
| **Primary tumor** | **Cellular DNA methylation** | | **Cell-free DNA methylation** | |
|  | ***SHOX2*** | ***SEPT9*** | ***SHOX2*** | ***SEPT9*** |
| **Digestive system** | 8/71 (30 %) | 17/71 (24 %) | 6/39 (15 %) | 14/39 (36 %) |
| Stomach | 2/6 (33 %) | 2/6 (33 %) | 2/4 (50 %) | 2/4 (50 %) |
| Small intestine | 0/2 (0 %) | 0/2 (0 %) | 0/1 (0 %) | 0/1 (0 %) |
| Colon* | 0/8 (0 %) | 1/8 (13 %) | 1/5 (20 %) | 2/5 (40 %) |
| Rectum | 0/2 (0 %) | 0/2 (0 %) | 0/1 (0 %) | 0/1 (0 %) |
| Anus, anal canal, & anorectum* | 0/1 (0 %) | 1/1 (100 %) | 0/1 (0 %) | 1/1 (0 %) |
| Liver & pancreas* | 4/31 (13 %) | 8/31 (26 %) | 2/13 (15 %) | 4/13 (31 %) |
| Gallbladder & bile ducts* | 2/21 (10 %) | 5/21 (24 %) | 1/14 (7 %) | 5/14 (36 %) |
|  |  |  |  |  |
| **Respiratory system** | 0/5 (0 %) | 0/5 (0 %) | 1/4 (25 %) | 0/4 (0 %) |
| Head and neck squamous cell carcinoma * | 0/2 (0 %) | 0/2 (0 %) | 1/2 (50 %) | 0/2 (0 %) |
| Lung & bronchus* | 0/3 (0 %) | 0/3 (0 %) | 0/2 (0 %) | 0/2 (0 %) |
|  |  |  |  |  |
| **Pleural mesothelioma** | 1/1 (100 %) | 0/1 (0 %) | - | - |
|  |  |  |  |  |
| **Melanoma skin** | 0/1 (0 %) | 0/1 (0 %) | - | - |
|  |  |  |  |  |
| **Bones and joints** | 0/1 (0 %) | 0/1 (0 %) | 0/1 (0 %) | 0/1 (0 %) |
|  |  |  |  |  |
| **Breast*** | 0/6 (0 %) | 1/6 (17 %) | 0/4 (0 %) | 1/4 (25 %) |
|  |  |  |  |  |
| **Genital system** | 2/22 (9 %) | 2/22 (9 %) | 3/15 (20 %) | 3/15 (20 %) |
| Uterine cervix and uterine corpus | 0/2 (0 %) | 0/2 (0 %) | - | - |
| Ovary* | 2/18 (11 %) | 2/18 (11 %) | 3/14 (21 %) | 3/14 (21 %) |
| Prostate* | 0/2 (0 %) | 0/2 (0 %) | 0/1 (0 %) | 0/1 (0 %) |
|  |  |  |  |  |
| **Urinary system** | 2/6 (33 %) | 2/6 (33 %) | 0/3 (0 %) | 0/3 (0 %) |
| Urinary bladder & renal pelvis* | 1/4 (25 %) | 1/4 (25 %) | 0/3 (0 %) | 0/3 (0 %) |
| Kidney | 1/2 (50 %) | 1/2 (50 %) | - | - |
|  |  |  |  |  |
| **Brain and other nervous system** | 0/1 (0%) | 0/1 (0%) | 0/1 (0%) | 0/1 (0%) |
|  |  |  |  |  |
| **Lymphoma** | 2/17 (12 %) | 1/17 (6 %) | 2/13 (15 %) | 0/13 (0 %) |
| Non-Hodgkin lymphoma* | 2/11 (18 %) | 1/11 (9 %) | 2/10 (20 %) | 0/10 (0 %) |
| Hodgkin lymphoma* | 0/2 (0 %) | 0/2 (0 %) | 0/1 (0 %) | 0/1 (0 %) |
| Myeloma* | 0/4 (0 %) | 0/4 (0 %) | 0/2 (0 %) | 0/2 (0 %) |
|  |  |  |  |  |
| **Other & unspecified primary sites** | 0/3 (0 %) | 1/3 (33 %) | 1/1 (100 %) | 1/1 (100 %) |

Patients indicated by "*" suffer from more than one primary tumor. For detailed information view Additional File 2.
