# Supplementary material for: Diagnostic and prognostic value of SHOX2 and SEPT9 DNA methylation and cytology in benign, paramalignant, and malignant ascites
Source: Clin Epigenetics. 2016 Mar 1;8:24. doi: 10.1186/s13148-016-0192-7 (PMC4774089; doi:10.1186/s13148-016-0192-7)
Supplement: Additional file 2: Table S2. — Specification of 25 cancer patients suffering from more than one primary tumor. Other existing primary tumors are listed for patients suffering from more than one primary tumor. (DOC 76 kb) [file 13148_2016_192_MOESM2_ESM.doc]

**Additional File 2:** **Specification of 25 cancer patients suffering from more than one primary tumor.**

| **Primary tumor** | **Second primary tumor** | **Third primary tumor** |
| --- | --- | --- |
| **Digestive System** |  |  |
| Colon | Pancreas |  |
| Colon | Breast |  |
| Anus, anal canal, & anorectum | Breast |  |
| Liver & pancreas | Stomach | Small intestine |
| Liver & pancreas | Colon |  |
| Liver & pancreas | Lung & bronchus |  |
| Gallbladder & bile ducts | Colon |  |
| Gallbladder & bile ducts | Melanoma skin |  |
| Gallbladder & bile ducts | Breast |  |
| Gallbladder & bile ducts | Prostate |  |
| Gallbladder & bile ducts | Penis |  |
|  |  |  |
| **Respiratory system** |  |  |
| Head and neck squamous cell carcinoma | Lung & bronchus |  |
| Lung & bronchus | Breast |  |
|  |  |  |
| **Breast** | Pancreas |  |
| **Breast** | Vulva |  |
|  |  |  |
| **Genital system** |  |  |
| Ovary | Colon | Breast |
| Ovary | Uterine cervix |  |
| Ovary | Uterine corpus |  |
| Ovary | Non-Hodgkin lymphoma |  |
| Prostate | Squamous cell carcinoma (nose) |  |
|  |  |  |
| **Urinary system** |  |  |
| Urinary bladder & renal pelvis | Liver |  |
| Urinary bladder & renal pelvis | Prostate |  |
|  |  |  |
| **Lymphoma** |  |  |
| Hodgkin lymphoma | Liposarcoma |  |
| Myeloma | Colon |  |
| Myeloma | Prostate |  |
